# Minimally invasive versus open distal pancreatectomy for pancreatic ductal adenocarcinoma (DIPLOMA): study protocol for a randomized controlled trial

**DOI:** 10.1186/s13063-021-05506-z

**Published:** 2021-09-09

**Authors:** Jony van Hilst, Maarten Korrel, Sanne Lof, Thijs de Rooij, Frederique Vissers, Bilal Al-Sarireh, Adnan Alseidi, Adrian C. Bateman, Bergthor Björnsson, Ugo Boggi, Svein Olav Bratlie, Olivier Busch, Giovanni Butturini, Riccardo Casadei, Frederike Dijk, Safi Dokmak, Bjorn Edwin, Casper van Eijck, Alessandro Esposito, Jean-Michel Fabre, Massimo Falconi, Giovanni Ferrari, David Fuks, Bas Groot Koerkamp, Thilo Hackert, Tobias Keck, Igor Khatkov, Ruben de Kleine, Arto Kokkola, David A. Kooby, Daan Lips, Misha Luyer, Ravi Marudanayagam, Krishna Menon, Quintus Molenaar, Matteo de Pastena, Andrea Pietrabissa, Rushda Rajak, Edoardo Rosso, Patricia Sanchez Velazquez, Olivier Saint Marc, Mihir Shah, Zahir Soonawalla, Ales Tomazic, Caroline Verbeke, Joanne Verheij, Steven White, Hanneke W. Wilmink, Alessandro Zerbi, Marcel G. Dijkgraaf, Marc G. Besselink, Mohammad Abu Hilal, for the European Consortium on Minimally Invasive Pancreatic Surgery (E-MIPS)

**Affiliations:** 1 Department of Surgery, Amsterdam UMC, University of Amsterdam, Cancer Center Amsterdam, VUMC, ZH-7F18, PO Box 7057, 1007 MB Amsterdam, the Netherlands; 2 Department of General Surgery, Instituto Ospedaliero Fondazione Poliambulanza, Brescia, Italy; 3 Department of Surgery, Morriston Hospital, Swansea, UK; 4 Department of Surgery, Virginia Mason Medical Center, Seattle, USA; 5 Department of Cellular Pathology, University Hospital Southampton NHS Foundation Trust, Southampton, UK; 6 Department of Surgery in Linköping and Department of Biomedical and Clinical Sciences, Linköping University, Linköping, Sweden; 7 Department of Surgery, Universitá di Pisa, Pisa, Italy; 8 Department of Surgery, Sahlgrenska University Hospital, Gothenburg, Sweden; 9 Department of Surgery, Pederzoli Hospital, Peschiera, Italy; 10 Division of Pancreatic Surgery IRCCS, Azienda Ospedaliero Universitaria Department of Internal Medicine and Surgery (DIMEC), S. Orsola-Malpighi Hospital, Alma Mater Studiorum, University of Bologna, Bologna, Italy; 11 Department of Pathology, Cancer Center Amsterdam, Amsterdam UMC, Amsterdam, the Netherlands; 12 Department of HPB surgery and liver transplantation, Beaujon Hospital, Clichy, France; 13 Department of Surgery, Oslo University Hospital and Institute for Clinical Medicine, Oslo, Norway; 14 Department of Surgery, Erasmus MC Cancer Institute, Rotterdam, the Netherlands; 15 Department of General and Pancreatic Surgery - Pancreas Institute, University Hospital of Verona, Verona, Italy; 16 Department of Surgery, Hopital Saint Eloi, Montpellier, France; 17 Department of Surgery, San Raffaele Hospital IRCCS, Università Vita-Salute, Milan, Italy; 18 Department of Surgery, Niguarda Ca’Granda Hospital, Milan, Italy; 19 Department of Surgery, Institut Mutualiste Montsouris, Paris, France; 20 Department of Surgery, Heidelberg University Hospital, Heidelberg, Germany; 21 Department of Surgery, UKSH campus Lübeck, Lübeck, Germany; 22 Department of Surgery, Moscow Clinical Scientific Center, Moscow, Russian Federation; 23 Department of Surgery, University Medical Center Groningen, Groningen, the Netherlands; 24 Department of Surgery, University of Helsinki and Helsinki University Hospital, Helsinki, Finland; 25 Department of Surgery, Emory University Hospital, Atlanta, USA; 26 Department of Surgery, Medisch Spectrum Twente, Enschede, the Netherlands; 27 Department of Surgery, Catharina Ziekenhuis, Eindhoven, the Netherlands; 28 Department of HPB Surgery, University Hospital Birmingham, Birmingham, UK; 29 Department of Surgery, King’s College Hospital NHS Foundation Trust, London, UK; 30 Department of Surgery, University Medical Center Utrecht, Utrecht, the Netherlands; 31 Department of Surgery, IRCCS Policlinico San Matteo Pavia, Pavia, Italy; 32 Department of Surgery, Hospital del Mar, Barcelona, Spain; 33 Department of Surgery, Centre Hospitalier Regional D’Orleans, Orleans, France; 34 Department of Surgery, Oxford University Hospital NHS Foundation Trust, Oxford, UK; 35 Department of Surgery, University Medical Center Ljubljana, Ljubljana, Slovenia; 36 Department of Pathology, University of Oslo, Oslo, Norway; 37 Department of Surgery, The Freeman Hospital Newcastle Upon Tyne, Newcastle, UK; 38 Department of Medical Oncology, Cancer Center Amsterdam, Amsterdam UMC, Amsterdam, the Netherlands; 39 Department of Surgery, Humanitas Clinical and Research Center-IRCCS, Rozzano (MI) and Humanitas University, Pieve Emanuele, MI Italy; 40 Department of Epidemiology and Data Science, Amsterdam UMC, University of Amsterdam, Amsterdam, the Netherlands; 41 Department of General Surgery, Fondazione Poliambulanza Instituto Ospedaliero, Brescia, Italy

**Keywords:** Minimally invasive, Laparoscopic, Robot-assisted, Distal pancreatectomy, Left pancreatectomy, Pancreatic tail resection, Pancreatic surgery, Pancreatic cancer, Pancreatic ductal adenocarcinoma

## Abstract

**Background:**

Recently, the first randomized trials comparing minimally invasive distal pancreatectomy (MIDP) with open distal pancreatectomy (ODP) for non-malignant and malignant disease showed a 2-day reduction in time to functional recovery after MIDP. However, for pancreatic ductal adenocarcinoma (PDAC), concerns have been raised regarding the oncologic safety (i.e., radical resection, lymph node retrieval, and survival) of MIDP, as compared to ODP. Therefore, a randomized controlled trial comparing MIDP and ODP in PDAC regarding oncological safety is warranted. We hypothesize that the microscopically radical resection (R0) rate is non-inferior for MIDP, as compared to ODP.

**Methods/design:**

DIPLOMA is an international randomized controlled, patient- and pathologist-blinded, non-inferiority trial performed in 38 pancreatic centers in Europe and the USA. A total of 258 patients with an indication for elective distal pancreatectomy with splenectomy because of proven or highly suspected PDAC of the pancreatic body or tail will be randomly allocated to MIDP (laparoscopic or robot-assisted) or ODP in a 1:1 ratio. The primary outcome is the microscopically radical resection margin (R0, distance tumor to pancreatic transection and posterior margin ≥ 1 mm), which is assessed using a standardized histopathology assessment protocol. The sample size is calculated with the following assumptions: 5% one-sided significance level (*α*), 80% power (1-*β*), expected R0 rate in the open group of 58%, expected R0 resection rate in the minimally invasive group of 67%, and a non-inferiority margin of 7%. Secondary outcomes include time to functional recovery, operative outcomes (e.g., blood loss, operative time, and conversion to open surgery), other histopathology findings (e.g., lymph node retrieval, perineural- and lymphovascular invasion), postoperative outcomes (e.g., clinically relevant complications, hospital stay, and administration of adjuvant treatment), time and site of disease recurrence, survival, quality of life, and costs. Follow-up will be performed at the outpatient clinic after 6, 12, 18, 24, and 36 months postoperatively.

**Discussion:**

The DIPLOMA trial is designed to investigate the non-inferiority of MIDP versus ODP regarding the microscopically radical resection rate of PDAC in an international setting.

**Trial registration:**

ISRCTN registry ISRCTN44897265. Prospectively registered on 16 April 2018.

## Background

Several randomized trials have suggested superiority of minimally invasive surgery over open surgery in terms of postoperative pain, morbidity, and length of hospital stay [1–5]. Minimally invasive distal pancreatectomy (MIDP), first described by Gagner in 1996 [6], is considered the standard approach for symptomatic benign and premalignant disease of the distal pancreas in many centers around the world [7, 8]. Although the number of pancreatic resections performed through a minimally invasive approach increased significantly during the past two decades, the initial introduction of minimally invasive pancreatic surgery has been rather slow [9, 10]. Recently, the first single- and multicenter, randomized, controlled trials comparing MIDP with open distal pancreatectomy (ODP) showed clear benefits of MIDP in terms of less intra-operative blood loss, a 2-day reduction in both time to functional recovery and length of hospital stay, and lower rates of delayed gastric emptying [5, 11]. However, these trials focused on MIDP for all indications (benign, premalignant, and malignant) and included only a minority of patients with pancreatic ductal adenocarcinoma (PDAC). Therefore, these trials were insufficient to draw any conclusions on the oncological outcome of the minimally invasive approach [5].

The oncological safety of MIDP remains a subject of debate which hampers its further implementation [12, 13]. A European survey on minimally invasive pancreatic surgery demonstrated that 73% (*n* = 148) of surgeons from 27 countries regularly performed MIDP [14]. Less than half of these surgeons, however, performed MIDP for PDAC and 31% expected MIDP to be inferior to ODP concerning oncological outcomes [14]. Consequently, many patients affected by PDAC might not receive MIDP due to uncertainty regarding oncological safety, and these patients do not benefit from the potential advantages. In theory, this enhanced recovery could potentially improve the use of adjuvant chemotherapy compared to ODP, as shown for other pancreatic resections [15–17].

Based on these results, the European Consortium on Minimally Invasive Pancreatic Surgery (E-MIPS) initiated the DIPLOMA study (distal pancreatectomy, minimally invasive or open, for malignancy). First, an international retrospective cohort study was performed including 1377 patients who underwent distal pancreatectomy (minimally invasive or open) for PDAC between 2007 and 2015 [18]. Patients after MIDP and ODP were matched using propensity scores in order to correct for standard demographics and known confounders. This study showed higher R0 resection rates for MIDP (67% versus 58%, *p* = 0.02), but lower median number of harvested lymph nodes [14 (interquartile range (IQR) 8–22) versus 22 (IQR 14–31), *p* < 0.001] with similar survival [29 (95% CI 23–35) versus 31 (95% CI 24–38) months for MIDP and ODP, respectively, *p* = 0.98] [18]. However, MIDP was associated with lower rates of lymphovascular tumor invasion, perineural tumor invasion, and lower lymph node stage insinuating that, despite matching, treatment allocation bias could still have influenced these results. Several other multicenter matched cohort studies on MIDP versus ODP for PDAC showed comparable outcomes, thus supporting the prevailing oncological uncertainty [19, 20].

The objective of the DIPLOMA trial is to compare the microscopically radical (R0) resection rate, survival, complications, quality of life, and costs after MIDP and ODP in patients with suspected or proven PDAC of the pancreatic body or tail. The outcome of the DIPLOMA trial will guide the further implementation of MIDP worldwide.

## Methods

### Design

The DIPLOMA trial is an international, randomized controlled, parallel-group, patient- and assessor (pathologist)-blinded non-inferiority trial comparing MIDP with ODP in patients with PDAC located in the pancreatic body or tail. Patients are randomly allocated to MIDP or ODP. Inclusion started after approval of the Medical Ethics Review Committee Board of Amsterdam UMC (location Academic Medical Center) in the Netherlands and the Research Ethics Committee of the University Hospital Southampton in the UK. Additionally, local approval was gained for every participating center. All patients provide a written informed consent before randomization. This protocol was developed according to the SPIRIT guidelines [21].

### Study population

**Fig. 1:**
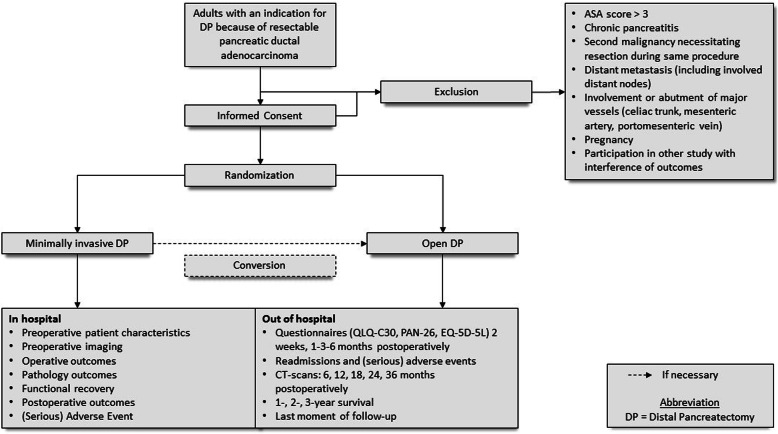
Study flowchart according to SPIRIT

**Fig. 2:**
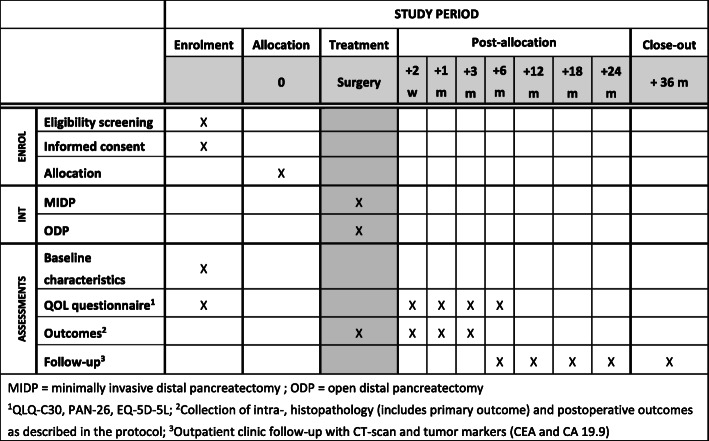
Schedule of enrolment, interventions, and assessments according to SPIRIT

Adult patients with an indication for elective distal pancreatectomy because of upfront resectable (proven or suspected) PDAC in the pancreatic body or tail are assessed for eligibility in the DIPLOMA trial (Figs. 1 and 2). PDAC is in this trial defined according to the WHO classification [22]. This may, albeit rare, also includes adeno-squamous carcinoma, colloid carcinoma (mucinous non-cystic carcinoma), hepatoid carcinoma, medullary carcinoma, signet ring cell carcinoma, undifferentiated carcinoma, and undifferentiated carcinoma with osteoclast-like giant cells.

### Inclusion criteria

In order to be eligible to participate in this study, a patient must meet all of the following criteria:

- Age of at least 18 years
- Elective indication for distal pancreatectomy for proven or suspected* PDAC
- Upfront (without induction/down-sizing radiotherapy and/or chemotherapy) resectable PDAC in the pancreatic body or tail^#^
- The tumor is expected to be radically resected via both MIDP and ODP according to the local treating team^$^
- The patient is fit enough to undergo MIDP and ODP according to the operating team

*Pathological proof is not mandatory for two reasons. First, it is not common practice in PDAC of the pancreatic body or tail; the decision for minimally invasive or open surgery will therefore after the trial also depend on the “suspected” diagnosis. Second, there are some concerns regarding the safety of endoscopic fine needle aspiration of distal pancreatic cancers with the theoretical risk of peritoneal seeding.

^#^Neoadjuvant radiotherapy and chemotherapy are allowed only in case of an upfront resectable tumor. Induction treatment for an initially non-resectable tumor (i.e., locally advanced) is not allowed.

^$^Extended resections are allowed, with the exclusion of vascular resections, if according to the local treating team, feasible with both a minimally invasive and open approach. Extended resections, including adrenal gland resection, are defined according to the ISGPS guideline [23]. The preoperative CT scan of every randomized patient will be crosschecked by an expert panel, blinded for including center and treatment allocation.

### Exclusion criteria

Patients meeting any of the following criteria will be excluded from participation in this study:

- American Society of Anesthesiology physical status above 3
- A medical history of chronic pancreatitis (according to the M-ANNHEIM criteria [24])
- Second malignancy necessitating resection during the same procedure
- Distant metastases (M1) including involved distant lymph nodes
- Tumor involvement or abutment of major vessels (celiac trunk*, mesenteric artery, or porto-mesenteric vein)
- Pregnancy
- Participation in another study with interference of study outcomes
- Malignant transformed pancreatic cystic lesion

*The distance between the tumor and the celiac trunk has to be at least 5 mm.

### Randomization

Patient recruitment and the collection of written informed consent are performed at the outpatient clinic. Allocation of patients to MIDP or ODP is performed centrally by the study coordinators using a concealed online computer-controlled permuted-block randomization module (Castor EDC, CIWIT B.V., Amsterdam, the Netherlands). Randomization between MIDP and ODP will be performed in a 1:1 ratio. The block sizes will be subject to random variation with block sizes varying between 4, 6, and 8 patients. The entire randomization will be concealed to all involved investigators. Randomization will be stratified for annual distal pancreatectomy volume (< 20, 20–40, and > 40) of participating centers. In addition, randomization will be stratified for tumor involvement beyond the pancreas and spleen to correct for differences in extended resections (adrenal gland involvement is defined as extended resection according to the ISGPS guideline) [23]. Randomization will take place as soon as distal pancreatectomy is planned (i.e., date of surgery is available) in order to reduce the drop-out rate after randomization. Only patients that do not receive surgery are considered a drop-out and will not be analyzed. All other randomized patients (including patients with peroperative diagnosed metastasized disease) will be analyzed according to the intention-to-treat principle. Patients will be coded by a numeric randomization code and the study coordinator will be the only one with access to it. The source data will be stored digitally and will be kept by the project leader for 15 years after the last patient’s follow-up is completed.

### Surgical technique

During several face-to-face meetings of the participating surgeons, agreement was reached regarding the (oncological) standards that should be followed during both minimally invasive and open distal pancreatectomy. Previously published and renowned standards for oncological distal pancreatectomy (formally called a left pancreatectomy) were followed; these standards were substantially described by Strasberg et al. [25] for the open approach and by Abu Hilal et al. [26] for the minimally invasive approach, including:

- Gerota’s fascia: routine resection of Gerota’s fascia should be performed in all patients.
- Splenectomy: should be performed in all patients.
- Lymphadenectomy: the ISGPS criteria for lymph node resection [27] will be followed. This includes resection of lymph node stations 10, 11, and 18 for tumors of the pancreatic body and tail. Additional resection of stations 8 and 9 will be performed when tumors affect the body of the pancreas. The aim is to resect a minimum of 11 lymph nodes.
- Transection of the pancreas: will be performed at the neck of the pancreas. The use of a stapling device is preferred but not mandatory as long as the same method is used in MIDP and ODP in each individual center.
- Transection of the splenic artery and vein: the use of clips (e.g., hem-o-lok, regular metal clips), staples, or sutures is not mandatory as long as the same method is used in MIDP and ODP in each individual center.
- Frozen sections: sampling of frozen sections is not mandatory, but surgeons should follow the same routine in both MIDP and ODP in each individual center.
- Surgical margins: sutures will be used to mark all surgical [transection (neck of the pancreas), posterior (Gerota’s fascia), and anatomical (anterior (peritonealised) and superior)] margins.

No specific other standards for MIDP and ODP are provided. Several of the above-mentioned details (e.g., regarding the method of transection of the pancreas and splenic artery and vein) are also relevant for the blinding of pathologists. All procedure details will be recorded within the case report form.

### Conversion from MIDP to ODP

Any incision used for other reasons than trocar placement or specimen extraction is defined as a conversion. Patients allocated to MIDP but converted to ODP will still be analyzed in the MIDP group, according to intention-to-treat principles. Reasons for conversion will be registered and categorized as urgent or non-urgent conversions [28].

### Blinding

Within the DIPLOMA trial, all assessors of the primary outcome (i.e., the pathologists), the patients, and the adjudication committee are blinded for treatment allocation. Directly after skin closure, while still under general anesthesia, patients will receive a firmly taped, large 40 × 40 cm abdominal dressing to cover their incision(s) and therefore their treatment allocation (minimally invasive or open). This abdominal dressing will be removed when all criteria for functional recovery are met or earlier for medical reasons, such as suspicion of wound infection. If earlier inspection is required, attempts are made to maintain patient blinding. This blinding has proven to be successful in previous multicenter trials [5, 29, 30]. The success of blinding will be assessed using the blinding index as proposed by Bang et al. [31]. Both patients and pathologists will be asked about the alleged treatment allocation, based on five categories: (1) strongly believe it was MIDP, (2) somewhat believe it was MIDP, (3) somewhat believe it was ODP, (4) strongly believe it was ODP, and (5) do not know. Due to ethical and legal concerns, patient blinding will not be performed in participating centers from the USA and in patients who are intra-operatively diagnosed with irresectable disease, such as metastases. Since patient blinding only influences one of the secondary outcomes, time to functional recovery, and not the primary outcome, this is considered of minor influence. Sensitivity analysis, excluding centers from the USA and patients with perioperative diagnosed metastasized disease will be performed for analysis of time to functional recovery.

### General treatment regimen

Because of the pragmatic design of the DIPLOMA trial, there are no restrictions regarding postoperative care, blood tests, drain management, the use of medication, or other kinds of co-intervention. However, the participating centers should provide the same postoperative care for both study arms (MIDP and ODP), based on enhanced recovery principles, which include early mobilization and expanding oral intake as desired by the patient. The treating team will be asked to specify the use of this kind of additional (surgical) proceedings and medication in the online case report forms.

### Primary outcome

The primary outcome is the microscopically radical resection margin (R0) (including the transection and posterior margins (surgical margins), but excluding the anterior and superior/inferior margins/surface (anatomical margins)). R0 is defined as a distance between the margin and the tumor of ≥ 1 mm [32]. A standardized histopathology assessment protocol and corresponding online webinar showing the standardized histopathological assessment methods has been developed by expert hepato-pancreato-biliary pathologists from large pancreatic surgery centers participating in the trial. Pathologists from all participating centers will complete this webinar before participation in the trial. This protocol is based on recent and relevant literature and was agreed on by all participating pathologists. Histopathological assessment within the DIPLOMA trial is only performed by the pathologists who completed the webinar. In order to ensure uniformity, study coordinators will be present in all centers during surgery of the first patient and subsequent handling of the specimen by the pathologist. Also, a validation will be performed by reviewing 10% of specimens by external pathologists. Involved pathologists will be blinded for the applied surgical approach.

### Secondary outcomes

The most important secondary outcomes are overall survival, time and site of disease recurrence, and postoperative time to functional recovery. Other secondary outcomes of this trial include intra-operative parameters (type of surgery (laparoscopic or robot-assisted), conversion, method of pancreatic transection, vessel resection, operative time, blood loss, and blood transfusion) and postoperative outcomes (major complications, delayed gastric emptying, pancreatic fistula, post-pancreatectomy hemorrhage, surgical site infection, serum levels of CA 19.9 and CEA, postoperative intervention, intensive care unit admission, organ failure, length of hospital stay, readmission, (time to) start of adjuvant therapy). Additional to the primary outcome, other pathology outcomes are recorded, such as tumor size, specimen length and weight, histology grading, distance from the tumor to all margins, number of retrieved lymph nodes, number of positive lymph nodes, lymphovascular and perineural tumor invasion, and venous and arterial tumor involvement. For the economical evaluation, costs (intra-operative and postoperative costs) and quality of life are assessed.

### Data collection and patient follow-up

The required clinical data will be collected after randomization, i.e., from hospitalization up to 36 months postoperatively using standardized (online) case report forms (Castor EDC, CIWIT B.V., Amsterdam, the Netherlands) by the local treating physicians. All data is stored in an electronic database (Castor EDC, CIWIT B.V., Amsterdam, the Netherlands). Data monitoring is performed by the study coordinators who will crosscheck the case report forms with source data. Baseline characteristics (age, sex, performance status (Karnofsky score)), ASA physical status, body mass index, previous abdominal surgery, diabetes mellitus, preoperative imaging conclusion including tumor size and involvement of other organs and vessels, neoadjuvant treatment, serum levels of Hba1C, CA 19.9 and CEA, and baseline quality of life measures will be recorded before randomization. For all patients, the most recent preoperative imaging will be sent anonymously to the study coordinator. This will follow local ethical and privacy rules in every center. After completion of the study, all imaging modalities will be reassessed by two expert radiologists independently in order to define the preoperative pancreatic cancer stages. In case of disagreement between the expert radiologists, a third expert radiologist will be invited and discussion will take place until consensus is reached. The required clinical data will be collected after randomization, i.e., from hospitalization up to 36 months postoperatively using standardized (online) case report forms by the local treating physicians, and will be crosschecked with source data by the study coordinators. For quality of life, the EQ-5D-5L, QLQ-C30, and PAN-26 questionnaires are used. These will be sent to the participating patients at baseline, 14, 30, 90, and 180 days after surgery. Patients will also receive a questionnaire at 90 days and 365 days after surgery which focuses on readmissions, complications, body image, and adjuvant therapy. Patients will be followed up at the outpatient clinic every 6 months during the first 2 years after surgery and after 36 months after surgery (i.e., at 6, 12, 18, 24, 36 months). During these follow-up moments, patients will undergo an abdominal CT scan and serum levels of CA 19.9 and CEA tumor markers will be assessed.

### Definitions

Functional recovery is reached when all of the following criteria are met: adequate pain control with only oral analgesia, restoration of mobility to a level of independence, ability to maintain sufficient caloric intake (a minimum of 50% of the required daily intake), no need for intravenous fluid administration and no signs of active infection (no fever or other clinical symptoms) [5, 29]. Complications are classified using the Clavien-Dindo score [33]. Major complications are defined as a Clavien-Dindo grade III or higher. Postoperative pancreatic fistula [34], delayed gastric emptying [35], and post-pancreatectomy haemorrhage [36] are classified the International Study Group on Pancreatic Surgery (ISGPS) definitions and only grade B and C complications will be recorded. Surgical site infection is classified according to the Centers for Disease Control and Prevention definition [37]. TNM staging is classified according to the American Joint Committee on Cancer (AJCC) classification (8th edition) [38].

### Quality and safety

Centers are allowed to participate in the DIPLOMA trial if they perform at least 15 distal pancreatectomies (any diagnosis) annually. Surgeons are allowed to participate if they have performed over 50 pancreatic resections (minimally invasive or open for any diagnosis), 50 advanced minimally invasive gastrointestinal resections (defined as any procedure beyond diagnostic laparoscopy, cholecystectomy, appendectomy, and inguinal hernia repairs), and 20 MIDPs for any diagnosis, including 5 for PDAC, and 10 ODPs for PDAC in the last 5 years. Surgeons that only perform open procedures in the trial should have performed over 50 pancreatic resections (for any diagnosis), 20 ODPs for any diagnosis, and 10 ODPs for PDAC in the last 5 years. All MIDP surgeons will be asked to send a recorded and anonymized video of a MIDP performed before the start of the trial. This video will be shortened and evaluated by an independent expert who is blinded for the surgeon and the clinical outcome. The videos are scored in five domains of technical skill (gentleness, tissue exposure, instrument handling, time and motion, and flow of the operation) using the methods described by Birkmeyer et al. [39]. Each domain will be rated on a scale of 1 to 5, where 1 indicates the skill expected of a general surgical resident and 5 the skill of a master minimally invasive pancreatic surgeon. A score of 3 points for every domain is considered an average minimally invasive pancreatic surgeon and should be scored in order to participate in the DIPLOMA trial. In addition, during operations (MIDP and ODP) within the DIPLOMA trial, surgeons will be asked to take photos of the pancreatic transection margin and the pancreatic bed (posterior margin) and send these to the trial coordinator, to objectify surgical quality.

All adverse events will be recorded up to 90 days postoperatively. Serious adverse events will be reported through a web portal (www.toetsingonline.nl) to the Dutch central committee on research involving human subjects (in Dutch: centrale commissie mensgebonden onderzoek) and the institutional review board (Medical Ethics Committee of Amsterdam UMC, location Academic Medical Center). Serious adverse events that have to be reported to the study coordinator within 24 h are unplanned intensive care unit admission; any surgical, endoscopic, or interventional radiology intervention (excluding feeding tube placement); readmission; and mortality (regardless of cause). The remaining adverse events are recorded in a yearly overview list. An independent data safety monitoring board (DSMB) is appointed to evaluate the study safety parameters. When each 50th included patient has completed 30 days of follow-up, the DSMB will meet in order to assess the safety parameters. This meeting may be either a telephone or video conference. The DSMB exists of two independent statisticians, one independent gastroenterologist, and two independent surgeons. One of the clinicians is appointed as the DSMB chairman and a second member as secretary. The minutes of these meetings will be sent to the institutional review board of the study by the study coordinator and the trial steering committee. The DSMB will not be blinded and will be fully informed on all SAEs. The DSMB can request a full report of specific study outcomes whenever required. The study coordinator and principal investigator will only be present during the start (open discussion) of the DSMB meeting to provide the data and provide background information.

### Ethics

The DIPLOMA trial will be conducted according to the principles of the Declaration of Helsinki (64th version, October 2013) and in accordance with the local laws and regulations, such as in the Netherlands the Medical Research Involving Human Subjects Act. The local principal investigator is responsible to adhere to local laws and regulations. The independent ethics review board of the Amsterdam UMC, location Academic Medical Center (Amsterdam, the Netherlands), and University Hospital Southampton NHS Foundation Trust (Southampton, UK) have both approved the study protocol. Furthermore, approval from all local ethics committees of participating centers was also obtained. The trial is registered in the ISRCTN registry: ISRCTN44897265.

### Statistical aspects

#### Sample size calculation

The DIPLOMA trial is designed as a non-inferiority trial, hypothesizing that in patients with pancreatic cancer the rate of microscopically radical (R0) resection rate of MIDP is non-inferior to ODP. Based on data collected for the retrospective DIPLOMA cohort study (1377 patients, matched based on propensity scores) [18], the sample size is calculated with the following assumptions: 5% one-sided significance level (*α*), 80% power (1-*β*), expected R0 rate in the open group of 58%, expected R0 resection rate in the minimally invasive group of 67%, and a non-inferiority margin of 7%. Based on these assumptions, a sample size of 226 patients (113 patients per arm) is required. Including 2.5% drop-out after randomization (patients who undergo no surgery after randomization) and 10% metastasized disease leads to a total number of patients to be randomized of 258 (129 per study arm).

#### Statistical analysis

Primary and secondary endpoints will be crosschecked with data from primary sources, and a blinded adjudication committee will check them against the used definitions. The primary endpoint (R0 resection rate) will be tested for non-inferiority using the chi-square test as described by Dunnett and Gent [40]. The distribution of variables will be determined using several plots (boxplot, Q-Q plot, and histogram) and the Kolmogorov-Smirnov, Shapiro-Wilk, and Levene’s tests as appropriate. For comparison of normally distributed continuous variables, the independent samples *t*-test will be used and values will be expressed as means with standard deviations. Continuous non-normally distributed variables will be compared using the Mann-Whitney *U* test and values will be expressed as medians with interquartile ranges. Categorical variables will be compared by chi-square or Fisher’s exact test as appropriate, and values will be expressed as proportions. A two-tailed *p*-value < 0.05 will be considered statistically significant. Where possible, risk ratios with 95% confidence intervals will be reported. For the primary study outcome, the lower limit of the two-sided 90% confidence interval of the difference in proportions will be reported and compared with the non-inferiority margin. Time to event endpoints, such as time to functional recovery, time to recurrence, and overall survival, will be calculated using Kaplan-Meier estimations. A Cox regression analysis will be performed to investigate predictors of postoperative survival. All parameters with a *p*-value < 0.1 in a univariable analysis are included in the multivariable Cox regression analysis. Multivariable logistic regression analyses are performed to determine predictors for primary and secondary study outcomes, for example, R0 resection, the occurrence of major complications, and postoperative pancreatic fistula. Predictors for receiving adjuvant chemotherapy will be assessed and additional “as treated” analysis will be performed. Subgroup analysis will be performed comparing outcomes for patients with and without neoadjuvant chemotherapy. Intra-operative details and primary endpoint of this study are expected to be complete. For patients who are lost to follow-up, a sensitivity analysis will be performed to determine best case and worst case scenarios. A detailed statistical analysis plan will be drafted prior to database lock. Despite all prior preventive measures taken, a complex international trial may evoke unforeseen situations after database lock that threaten data integrity and can only be resolved by unlocking the database prior to the final analysis. For purpose of transparency and reproducibility, the statistical analysis plan will therefore also describe the procedure to be followed when such situations arise.

### Dissemination policy

The results of this trial will be submitted to a peer-reviewed medical journal regardless of the study outcome. Authorship will be based on international guidelines. Those involved with the study who do not fulfill these criteria will be listed as “collaborator.” As soon as the trial outcomes are available, all participating patients will receive a letter with a summary of the outcomes.

## Discussion

The DIPLOMA trial is an international randomized controlled, patient- and assessor (pathologist)-blinded trial assessing the non-inferiority of MIDP compared to ODP regarding the R0 resection rate in patients with PDAC. DIPLOMA was initiated by the European Consortium on Minimally Invasive Pancreatic Surgery (E-MIPS) and is the first international trial on minimally invasive pancreatic surgery and the first to compare oncological outcomes after MIDP and ODP for PDAC.

The World Health Organization trial registry, which incorporates all international trial registries, currently (search: August 26, 2020) includes five other trials comparing MIDP (or laparoscopic distal pancreatectomy only) with ODP. One of these trials was early terminated due to lack of funding without reporting of results (NCT00988793). Two trials are currently recruiting, first, the DISPACT-2 trial, compares laparoscopic with open distal pancreatectomy regarding complications according to the comprehensive complication index. This trial has a total sample size of 294 patients with benign, premalignant, and malignant disease (DISPACT-2, DRKS00014011). The second trial compares laparoscopic with open distal pancreatectomy for pancreatic cancer. The total sample size is 306 patients and the primary outcome is recurrence-free survival (NCT03792932).

Two trials on MIDP vs ODP have been published. The multicenter Dutch LEOPARD trial compared minimally invasive with open distal pancreatectomy [5]. The second completed trial is the monocenter Swedish LAPOP trial which compared laparoscopic with open distal pancreatectomy [11]. Both trials showed shorter time to functional recovery and shorter hospital stay after the minimally invasive/laparoscopic approach [5]. Important differences between the DIPLOMA trial and the published trials are the inclusion criteria and the primary outcome. Whereas the DIPLOMA trial specifically focuses on patients with PDAC of the pancreatic body and tail, the LEOPARD and LAPOP trial included all indications for resection (i.e., also benign and premalignant lesions). The primary outcome of the LEOPARD trial was time to functional recovery and the LAPOP trial length of hospital stay whereas the DIPLOMA trial focuses on oncological outcome, with the R0 resection rate as a primary outcome and survival as the most important secondary outcome. Furthermore, time to functional recovery and quality of life will also be studied during the DIPLOMA trial. In this way, the DIPLOMA trial will assess the oncological non-inferiority of MIDP but will, as a secondary outcome, also possibly confirm the results of the LEOPARD and LAPOP trial in patients with pancreatic cancer. To decrease the influence of bias, pathologists will be blinded for the approach during specimen assessment and completion of the case report forms. In addition, patients will be blinded as was done in the LEOPARD trial [5, 41].

The most relevant outcome for patients undergoing distal pancreatectomy for PDAC would be long-term survival. However, a non-inferiority trial with survival as a primary outcome would require over 10,000 patients, which is not considered feasible. Therefore, the microscopically radical resection (R0) rate was chosen as the most relevant “surrogate” outcome. The strong association between microscopically radical resection and survival has been shown extensively [42–47]. Nevertheless, R0 rates reported in previous randomized controlled trials vary widely [48]. This is probably related to the varying pathology assessment and used definitions of R0 resection (no involvement of the margin or a distance between the margin and the tumor of at least 1 mm). Since no specific pathology assessment and reporting guidelines or protocols for distal pancreatectomy are available, the interpretation of R0 may vary between centers or even between pathologists. To minimize this heterogeneity in the DIPLOMA trial, a specific pathology assessment and reporting protocol for pancreatic body and tail specimens was developed [49]. In addition, a webinar explaining all details of this protocol including pictures and videos was developed. Participating pathologists will complete this webinar prior to inclusion of patients in their center. To reduce potential bias towards MIDP or ODP, pathologists will be blinded for treatment allocation.

Uniformity of surgical technique is an important challenge in surgical trials [50]. During several meetings, the standards for surgical technique in the DIPLOMA trial were discussed extensively and ultimately agreed upon. Several steps of the standard technique could influence the oncological outcome and subsequently the primary outcome of the trial. Whenever possible and available, existing guidelines were followed [26, 27, 49]. Since current literature regarding the benefit of performing a radical antegrade modular pancreato-splenectomy (RAMPS) procedure (for ODP) and laparoscopic radical left pancreato-splenectomy is limited, this full procedure was not included as part of the standardized technique, but it was agreed to attain to the described techniques as much as possible. However, the decision was made to include resection of Gerota’s fascia as a standard step and most importantly to adhere to the same technique for both MIDP and ODP [25, 51, 52].

## Conclusion

In conclusion, the DIPLOMA trial is an international randomized controlled, patient- and assessor (pathologist)-blinded trial, designed to assess the non-inferiority of MIDP vs. ODP for the microscopically radical (R0) resection rate of PDAC. Potentially, if the oncological non-inferiority of MIDP is confirmed, DIPLOMA will further increase the implementation of MIDP and in this way improve patient outcomes.

## Trial status

Confirmation of funding of the trial by Covidien AG (Medtronic, Neuhausen am Rheinfall, Switzerland) was received on November 15, 2017. Ethical approval in the Amsterdam UMC was received on February 01, 2018, and in University Hospital Southampton NHS Foundation Trust on February 20, 2018. The DIPLOMA trial was registered in the ISRCTN registry on April 16, 2018 (ISRCTN44897265). The current used protocol is version 3 (September 2018). The following items were included in the protocol amendments: (1) additional centers were added, further specification of blinding of the pathologist; (2) preoperative radiotherapy was removed as an exclusion criterion (before the start of the trial); and (3) three additional questionnaires (an additional quality of life questionnaire at 14 days postoperative and additional questionnaires on readmissions, complications, body image and adjuvant therapy at 3 and 12 months after surgery).

The first patient was randomized on May 03, 2018. At the time of submitting this protocol for publication (January 12, 2021), all centers were actively recruiting patients for the trial and 217 out of 258 (84%) have been randomized, which means that inclusion is on schedule.

## Data Availability

Data will be available to members of E-MIPS. All publications and presentation using this data will be approved by all authors.

## Abbreviations

MIDP: Minimally invasive distal pancreatectomy
ODP: Open distal pancreatectomy
R0: Microscopically radical resection
PDAC: Pancreatic ductal adenocarcinoma
E-MIPS: European Consortium on Minimally Invasive Pancreatic Surgery
